# Negative Clusters Associated With Cannabis Use: Tangled Up in Blues

**DOI:** 10.1093/geroni/igab046.1969

**Published:** 2021-12-17

**Authors:** Hyojung Kang

**Affiliations:** University of Illinois, University of Illinois, Illinois, United States

## Abstract

Previous studies concerning older adults have focused on whether cannabis use leads to positive or negative outcomes. In this study, we identified clusters of negative health outcomes associated with medical cannabis use. In total, we examined eight health outcomes: pain, sleep, falls, memory, digestive issues, mental health conditions, exercise, and general productivity reported by 2,968 persons over 60 who participated in the Illinois Medical Cannabis Program. We used association analysis to simultaneously identify groups of negative outcomes reported by participants. The distribution of non-positive outcomes shows a bell-shaped curve: 1.4% of participants responded that cannabis use improved all outcomes, while 4.1% of participants answered that cannabis use did not. When looking at negative outcomes, 86% of participants reported none worsened, and 11% reported one of the outcomes was affected. Only a small fraction of the participants (3%) claimed more than one negative outcomes after cannabis use.

